# The EGF/EGFR axis and its downstream signaling pathways regulate the motility and proliferation of cultured oral keratinocytes

**DOI:** 10.1002/2211-5463.13653

**Published:** 2023-06-04

**Authors:** Ryota Kobayashi, Emi Hoshikawa, Taisuke Saito, Orakarn Suebsamarn, Eriko Naito, Ayako Suzuki, Seiichiro Ishihara, Hisashi Haga, Kei Tomihara, Kenji Izumi

**Affiliations:** ^1^ Division of Biomimetics, Faculty of Dentistry & Graduate School of Medical and Dental Sciences Niigata University Japan; ^2^ Division of Oral and Maxillofacial Surgery, Faculty of Dentistry & Graduate School of Medical and Dental Sciences Niigata University Japan; ^3^ Division of Periodontology, Faculty of Dentistry & Graduate School of Medical and Dental Sciences Niigata University Japan; ^4^ Product Development Department Photonic Lattice Inc Sendai Japan; ^5^ Children's Oral Health Department, Institute of Dentistry Suranaree University of Technology Nakhon Ratchasima Thailand; ^6^ Department of Advanced Transdisciplinary Sciences, Faculty of Advanced Life Science Hokkaido University Sapporo Japan

**Keywords:** EGF/EGFR axis, motility, oral keratinocyte, pharmacological manipulation, proliferation, quality control

## Abstract

We previously reported that the cell and colony motion of oral keratinocytes are correlated with proliferative capacity, and speculated that this may be a specific index for monitoring cell quality. However, how cell motility and proliferation are regulated by signaling pathways remains unelucidated. Here, we found that the regulation of cell motility and proliferative capacity of oral keratinocytes can be attributed to the epidermal growth factor/epidermal growth factor receptor (EGF/EGFR) axis. The EGFR downstream cascade involving the Src/PI3K/Akt/mTOR signaling pathway showed a major effect on cell motility and proliferative capacity in oral keratinocytes. Furthermore, both EGFR and Src attenuated E‐cadherin expression. Taken together, these findings provide a potential basis for future quality control of cells for therapeutic use.

## Graphical abstract

Knowledge of which signals are involved in oral keratinocyte cell motility and proliferative capacity remains limited. The EGF/EGFR axis and its downstream Src/PI3K/Akt/mTOR signaling pathway were demonstrated to regulate the motogenic and mitogenic activities of oral keratinocytes in our culture system. Additionally, EGFR and Src activation correlate with the expression of E‐cadherin, associated with a characteristic growth pattern of cells.

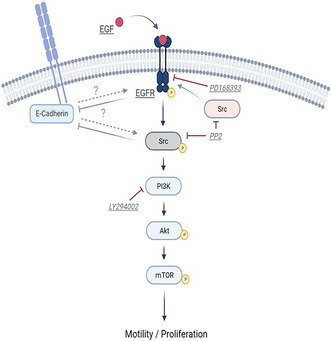

**Abbreviations**
18s‐rRNA: 18s‐ribosome ribonucleic acid
BSA: bovine serum albumin
cDNA: complementary deoxyribonucleic acid
DMSO: dimethyl sulfoxide
ECL: enhanced chemiluminescence
EDGS: EpiLife Defined Growth Supplements
EDTA: ethylenediaminetetraacetic acid
EGF: epidermal growth factor
EGFR: epidermal growth factor receptor
FAK: focal adhesion kinase
GAPDH: glyceraldehyde‐3‐phosphate dehydrogenase
HCS: hydrocortisone
HIV: human immunodeficiency virus
HRP: horseradish peroxidase
IGF‐1: insulin‐like growth factor I
IGF1R: insulin‐like growth factor I receptor
MAPK: mitogen‐activated protein kinase (MEK, ERK)
MMS: mean motion speed
mTOR: mammalian target of rapamycin
OF: optical flow
OKCs: oral keratinocytes
p130^Cas^
: p130 Crk‐associated substrate
PCR: polymerase chain reaction
PDT: population doubling time
PGE_2_
: prostaglandin E_2_
PI3K: phosphatidylinositol‐3 kinase
QC: quality control
RNA: ribonucleic acid
SDS: sodium dodecyl sulfate
SFKs: Src family kinases
siRNA: small interfering ribonucleic acid
STAT3: signal transducer and activator of transcription 3
TBST: Tris‐buffered saline with Tween 20
TF: transferrin
TFRC: transferrin receptor

Autologous oral mucosal epithelial cell sheets have been transplanted extraorally as an alternative therapeutic modality [1, 2, 3, 4, 5], and consequently, oral keratinocytes have become one of the major cell sources used in regenerative medicine. The maintenance of a sufficient number of keratinocyte stem cells, such as p63‐expressing limbal cells, is crucial for successful clinical translation [6]. Therefore, monitoring the quality of cultured keratinocytes during cell expansion and the manufacturing of epithelial cell sheets has a critical impact on clinical outcomes [7, 8]. However, to date, no reliable stem cell markers for oral keratinocytes have yet been identified [9, 10]. Existing analyses are reliable, but most entail destruction or damaging of cells, which directly compromises cells and cell‐based devices.

Recently, for skin keratinocytes co‐cultured on a mouse feeder layer, a novel noninvasive quality control (QC) technology was developed to predict the quality of keratinocyte stem cell populations [11, 12]. Accordingly, the motility of skin keratinocyte stem cells in holoclones, which is regulated by epidermal growth factor receptor (EGFR), was found to be positively correlated with their proliferative capacity. Therefore, cell motility appears to be a useful parameter for the noninvasive evaluation of keratinocyte stem cell populations. Nonetheless, because oral keratinocytes are cultured in a serum‐ and feeder layer‐free culture system, and present a scattered, non‐clonal growth pattern [13, 14, 15], we previously developed a different noninvasive and quantitative evaluation tool that uses an image‐based algorithm to evaluate cell motility. Our optical flow (OF)‐mediated noninvasive imaging technology is thus a useful tool for evaluating the cells/colony motion, since it provides an index of cell motility to monitor the quality of oral keratinocytes. We then found that the cells/colony motion was correlated with proliferative capacity during cell expansion [16]. However, the underlying molecular mechanisms regulating the cell motility associated with proliferation in oral keratinocytes have not yet been elucidated.

The EGFR signaling pathway plays a vital role in several important biological events of epithelial cells, including cell proliferation, survival, and migration [12, 17]. When specific ligands or extracellular signals, such as EGF, bind to EGFR, a number of its downstream signaling cascades are activated [18, 19]. Since the EGF/EGFR axis enables the massive expansion of human skin keratinocytes, allowing treatment of extensive burns and genetic disorders [20, 21], understanding the molecular mechanisms involved in cell motility and proliferation will be beneficial for the advancement of regenerative medicine using oral keratinocytes.

Here, we investigated the intracellular signaling regulating the cell motility and proliferative capacities of oral keratinocytes. The objectives of this study were to determine whether components of the growth supplements have a major effect on cells/colony motion and proliferative capacity, and to explore and characterize the intracellular signaling dynamics of EGFR and its downstream cascade. The resolution of a specific signaling pathway can provide potential targets for future therapies or pharmacological manipulations, offer novel insight into the molecular basis of the proliferation of oral keratinocytes, and help guide more efficient maintenance of keratinocyte progenitor/stem cell populations.

## Materials and methods

### Reagents and antibodies

All reagents and antibodies purchased for this study are listed in Table 1.

**Table 1:** List of reagents and antibodies used in this study.

Reagents	Vendors	Catalog number	Working Concentration
BSA	FujiFilm Wako pure chemical	015‐23871	30 μg per ML
hEGF	Lonza	CC‐4107	1 ng per ML
Transferrin	Lonza	CC‐4205	30 μg per ML
Hydrocortisone	FujiFilm Wako pure chemical	086‐10191	11 ng per ML
Insulin‐like growth factor‐1	Sigma‐Aldrich	I3769	10 ng per ML
Prostaglandin E_2_	Sigma‐Aldrich	P0409	18 ng per ML
PD168393	Selleck chemicals	S7039	40 nm
PP2	EMD Millipore Corp	529573	2 μm
U0126	EMD Millipore Corp	662005	200 nm
LLL12	BioVision	1792	200 nm
LY294002	Abcam	ab120243	2 μm
PF‐562271	Sigma‐Aldrich	PZ0387	3 μm
EGFR (Phospho‐Try1092) Antibody	Signalway Antibody LLC	#11081	1 : 500
EGF Recepter (C38B1) XP^®^ Rabbit mAb	Cell Signaling Technology	#4267	1 : 1000
Phospho‐Src Family (Tyr416) (D49G4) Rabbit mAb	Cell Signaling Technology	#6943	1 : 1000
Src (32G6) Rabbit mAb	Cell Signaling Technology	#2123	1 : 1000
Phospho‐Stat3 (Tyr705) (D3A7) XP^®^ Rabbit mAb	Cell Signaling Technology	#9145	1 : 2000
Stat3α (D1A5) XP^®^ Rabbit mAb	Cell Signaling Technology	#8768	1 : 1000
Phospho‐p44/42 MAPK (Erk1/2) (Thr202/Tyr204) (D13.14.4E) XP^®^ Rabbit mAb	Cell Signaling Technology	#4370	1 : 2000
p44/42 MAPK (Erk1/2) Antibody	Cell Signaling Technology	#9102	1 : 1000
Phospho‐Akt (Ser473) Antibody	Cell Signaling Technology	#9271	1 : 1000
Akt Antibody	Cell Signaling Technology	#9272	1 : 1000
Phospho‐mTOR (Ser2448) Antibody	Cell Signaling Technology	#2971	1 : 1000
mTOR Antibody	Cell Signaling Technology	#2972	1 : 1000
E‐Cadherin (24E) Rabbit mAb	Cell Signaling Technology	#3195	1 : 1000
β‐Actin (13E5) Rabbit mAb	Cell Signaling Technology	#4970	1 : 1000
Anti‐FAK (phopho Y397) antibody [EP2160Y]	Abcam	ab81298	1 : 1000
ECL™ Anti‐rabbit IgG	GE Healthcare	NA934‐1ML	0.2 μL per ML
ECL™ Anti‐mouse IgG	GE Healthcare	NA931‐1ML	0.2 μL per ML

### Procurement of oral mucosa samples

The protocol for obtaining human oral mucosa tissue samples was approved by the Niigata University Hospital Internal Review Board (Approval#2015‐5018). Patients who underwent minor dentoalveolar surgery were provided with sufficient information regarding this study, and all participating individuals signed an informed consent form prior to participation. Exclusion criteria based on which the participants were selected included as follows: (a) positive for infection with syphilis, HIV, or Hepatitis B or C virus using serological tests; (b) therapeutically uncontrolled diseases such as diabetes; (c) immunodeficiency; and (d) patients receiving radiation therapy. Samples of oral mucosal tissues were harvested from 12 individuals, which included four males and eight females, 17 to 43 years of age, with a mean value of 28.4 ± 7.3 years. The methodologies employed in this study conformed to the standards set by the Declaration of Helsinki.

### Primary oral keratinocytes cell culture

Primary oral keratinocytes were first cultured and multiplied using a previously described protocol [16]. Briefly, specimens were soaked in a 0.025% trypsin/ethylenediaminetetraacetic acid (EDTA) solution (Thermo Fisher Scientific, Waltham, MA, USA) containing 1.5% Antibiotic‐Antimycotic cocktail (Thermo Fisher Scientific) overnight at room temperature. Oral keratinocytes were then mechanically dissociated in 0.0125% defined trypsin inhibitor (Thermo Fisher Scientific), plated as p0 cells at a density of 4.0–5.0 × 10^4^ cells·cm^−2^, and fed with ‘complete’ EpiLife^®^ supplemented with EpiLife Defined Growth Supplements (EDGS; Thermo Fisher Scientific), 0.06 mm Ca^2+^, gentamicin (5.0 μg·mL^−1^; Thermo Fisher Scientific), and amphotericin B (0.375 μg·mL^−1^; Thermo Fisher Scientific) every other day. When p0 cells were approximately 80% confluent, they were subcultured and serially passaged. p2 to p5 cells were used in this study.

### Culture medium component analysis, time‐lapse imaging, and image processing

For culture medium component analysis, six EDGS components were purchased independently (Table 1). EpiLife^®^ supplemented with 30 μg·mL^−1^ BSA, gentamicin, and amphotericin B was prepared as the ‘basal medium’. Subsequently, we added one of the five EDGS components to the basal medium and withdrew one of the EDGS components from the ‘complete’ EpiLife^®^. In total, we obtained 12 culture media formulations, including ‘basal’ and ‘complete’ media. The different culture media were then used to analyze the effect of individual EDGS components on cells/colony motion and proliferative capacity (*n* = 7). These trials were conducted as follows: first, 5.0–7.0 × 10^4^ cells were plated onto 12 wells of 6‐well microplates and cultured in ‘complete’ EpiLife^®^ for 24 h. This medium was then replaced with one of the 12 different test media, in which the final concentration of the individual component was as follows: 30 μg·mL^−1^ for TF, 11 ng·mL^−1^ for HCS, 10 ng·mL^−1^ for IGF‐1, 18 ng·mL^−1^ for PGE_2_, and 1 ng·mL^−1^ for EGF, respectively. When cells cultured in ‘complete’ EpiLife^®^ were approximately 50–60% confluent, randomly chosen six locations in each of 12 wells were micro‐photographed at 15‐min intervals for 24 h using a CFI Plan Fluor DL4× objective lens equipped on a BioStudio‐T imaging platform (Nikon, Tokyo, Japan; *n* = 7).

### Determination of cell motility and proliferative capacity

A total of 96 images were converted to video files using cell image viewer2 (Nikon), and we then determined the motility of cells cultured in each of the 12 test media conditions using of (Optical flow) algorithm‐based software [16]. For each condition, the average of the cell motility of the six locations was represented as the mean motion speed (MMS; measured in μm·h^−1^) of the sample (*n* = 7). To determine proliferative capacity, regardless of confluency we counted the number of cells harvested 24 h after the completion of time‐lapse imaging to determine population doubling time (PDT) [16].

### Determination of cell motility using siRNA treatment

Next, we used targeted gene silencing of EGFR, TFRC, and IGF1R using TaqMan^®^ gene expression assays (Thermo Fisher Scientific); this method was performed as described in our previous study [14]. siRNA‐EGFR (Silencer Select^®^ s565 (epidermal growth factor receptor)) (sense, GAUCUUUCCUUCUUAAAGAtt; antisense, UCUUUAAGAAGGAAAGAUCat), siRNA‐TFRC (Silencer Select^®^ s727 (transferrin receptor)) (sense, GGUCAUCAGGAUUGCCUAAtt; antisense, UUAGGCAAUCCUGAUGACCga), siRNA‐IGF‐1R (Silencer Select^®^ s7212 (insulin growth factor‐1 receptor)) (sense, CCGAAGAUUUCACAGUCAATT; antisense, UUGACUGUGAAAUCUUCGGCT), siRNA‐GAPDH, and negative control siRNA were purchased from Thermo Fisher Scientific.

As in our previous study [14], TaqMan^®^ gene expression assays (Thermo Fisher Scientific) were used for relative quantification. Analyses were conducted using an Applied Biosystems StepOnePlus Real‐Time PCR System (Applied Biosystems, Waltham, MA, USA). Total RNA was first extracted from cell pellets obtained from oral keratinocytes treated with previously listed siRNA reagents using a RNeasy^®^ Kit (Qiagen, 74104 Valencia, CA, USA). cDNA was generated by reverse transcription using a High‐Capacity RNA to cDNA kit (Thermo Fisher Scientific) with all procedures performed according to the manufacturer's protocol. Relative quantification analysis of EGFR (Hs01076090_m1), TFRC (Hs00951083_m1), and IGF1R (Hs00609566_m1) of cells was carried out using TaqMan^®^ gene expression assays. Target gene expression was normalized to the expression of the reference gene, 18s‐rRNA (Hs99999901_s1).

We then plated 3.0 × 10^4^ of oral keratinocytes onto 6 wells in a 12‐well microplate with ‘complete’ EpiLife^®^. When cells were 50% confluent, they were transfected with siRNA‐EGFR, TFRC, IGF‐1R, or siGAPDH, a negative control siRNA, without any reagent (*n* = 3). siRNA‐EGFR (Silencer Select^®^ s565 [epidermal growth factor receptor]) (Thermo Fisher Scientific), siRNA‐TFRC (Silencer Select^®^ s727 [transferrin receptor]), siRNA‐IGF‐1R (Silencer Select^®^ s7212 [insulin growth factor‐1 receptor]), siRNA‐GAPDH, and negative control siRNA were used in this study. Transfection was performed under ‘complete’ EpiLife^®^ without gentamicin and amphotericin B using Lipofectamine RNAi MAX (Thermo Fisher Scientific) with all methods performed as per the manufacturer's instructions. Eighteen hours later, randomly chosen five locations in each of six wells were micro‐photographed at 15‐min intervals for 24 h, yielding a total of 96 images. These were then converted to video files, and the MMS (μm·h^−1^) for each of the five locations was calculated as stated above (*n* = 3). Pellets of oral keratinocytes treated with the siRNA reagents mentioned in the previous paragraph were collected. Subsequently, the total RNA was extracted using an RNeasy^®^ Kit (Qiagen). cDNA was generated through reverse transcription using a High‐Capacity RNA to cDNA kit (Thermo Fisher Scientific). All procedures were performed according to the protocol recommended by the manufacturer.

### Determination of cell motility and proliferative capacity by pharmacological inhibitor assay

All inhibitors were dissolved in DMSO (Sigma‐Aldrich, St. Louis, MO, USA) according to the manufacturer's instructions. The concentrations of the pharmacological inhibitors used in this study were as follows: PD168393 (EGFR inhibitor): 40 nm, LY294002 (PI3K/Akt inhibitor): 2 μm, PP2 (Src inhibitor): 5 μm, LLL12 (STAT3 phosphorylation inhibitor): 200 nm, U0126 (MEK1/2 inhibitor): 200 nm, and PF‐562271 (Focal adhesion kinase (FAK) inhibitor): 3 μm. These concentrations were predetermined by the previously described cell viability assay [15], and none fell below a threshold of 95% of the absorbance of a vehicle (DMSO) control. For each inhibition experiment, 5.0–7.0 × 10^4^ cells were first plated onto 6‐well microplates and cultured with ‘complete’ EpiLife^®^ for approximately 24 h (*n* = 10). The medium was then replaced with the basal medium either with or without EGF (1 nm). After another incubation for 24 h, each specific inhibitor was added at the indicated concentration to medium containing EGF. After 36 h (or more), the randomly chosen six locations in the wells were photographed by time‐lapse microscope, and cell motility was determined using the method referenced above. The mean cell motility value of the six measured locations represented the MMS (μm·h^−1^) of the sample (*n* = 10). Regardless of confluency, PDT was determined 24–48 h after time‐lapse imaging.

### Western blot analysis

We then used western blots to measure protein expression levels. First, cells (*n* = 5) were washed three times with cold phosphate‐buffered saline and then lysed with 200 μL of lysis buffer (1.0 m Tris–HCl, 4% SDS, 2% glycerol, 10% 2‐Mercaptoethanol, and 0.005% bromophenol blue, adjusted to pH 6.8). The cell lysates were then ultra‐sonicated and centrifuged at 12 000 *g* for 5 min. Next, the supernatant was collected and quantified using a BIO‐RAD Protein Assay Kit (BIO‐RAD, Hercules, CA, USA), before denaturation at 100 °C for 10 min, after which all samples were stored at −80 °C for further use. Protein samples were then separated on 7.5%, 10%, or 12% polyacrylamide gels (BIO‐RAD) and then transferred to polyvinylidene difluoride membranes (BIO‐RAD). The non‐specific binding sites of all membranes were blocked using 5% non‐fat dry milk or 3% BSA (Sigma‐Aldrich) for 1 h at room temperature and then incubated overnight at 4 °C with the following (anti‐) primary antibodies: p‐EGFR (Tyr1092), EGFR, p‐Src family (Tyr416), Src, p‐Stat3 (Tyr705), Stat3α, p‐MAPK(ERK1/2) (Thr202/Tyr204), MAPK(ERK1/2), p‐Akt (Ser473), Akt, p‐mTOR (Ser2448), mTOR, p‐FAK, FAK, E‐cadherin, or β‐actin. After primary incubation, all membranes were washed with Tris‐buffered saline containing 0.05% Tween 20 (TBST) and then incubated with HRP‐conjugated secondary antibodies for 2 h at room temperature. Protein bands were visualized using western ECL substrate (BIO‐RAD), and images were acquired using chemidoc xrs Plus + image lab software (BIO‐RAD). β‐actin expression was used as a loading control. For E‐cadherin expression, the band intensity of representative gels was quantified and normalized to β‐actin. Densitometry values represent the ratio of expression levels of cells cultured in the basal medium.

### Statistical analyses

All statistical analyses were performed using graphpad prism 8 (GraphPad Software, San Diego, CA, USA). Differences in mean value among six (i.e., for medium component analysis and siRNA treatment) or three groups (i.e., for inhibitor assays) were analyzed using one‐way ANOVA, followed by Tukey's *post hoc* tests. Differences were considered statistically significant if *P*‐values were less than 0.05.

## Results

### Identification of a single growth supplement that regulates both cell motility and proliferation

**Fig. 1:**
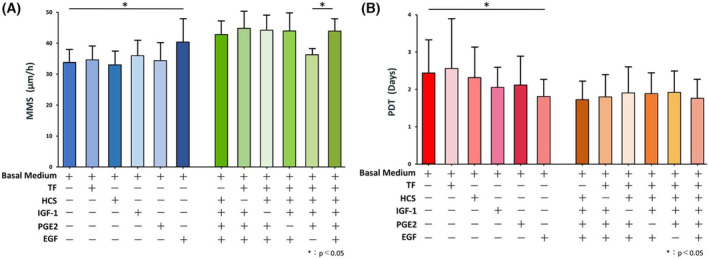
Determination of growth supplements regulating cell motility and proliferation by culture medium component analysis. (A) Mean MMS (mean motion speed: an index of cell motility: *N* = 7) values for oral keratinocytes cultured in different media. Shown are values for the basal medium plus a single growth supplement additive (left side) and a complete culture medium containing removal of a single growth supplement (right side). The addition of EGF to the basal medium and the removal of EGF from the complete culture medium significantly increased and decreased MMS (mean motion speed), respectively. Data are shown as mean ± SD. Significant differences among the groups were determined by one‐way ANOVA with Tukey's *post hoc* tests. *P < 0.05. (B) Mean PDT (population doubling time: an index of proliferative capacity: *N* = 7) values for oral keratinocytes cultured in different media. Shown are values for the basal medium plus a single growth supplement additive (left side) and a complete culture medium containing removal of a single growth supplement (right side). The addition of EGF to the basal medium significantly decreased PDT (population doubling time). Data are shown as the mean ± SD. Significant differences among the groups were determined by one‐way ANOVA with Tukey's *post hoc* tests. *P < 0.05.

We first examined which component of the growth supplements examined showed a major effect on cells/colony motion and the proliferative capacity of oral keratinocytes by comparing their relative MMS and PDT parameters, respectively. MMS showed a significant increase when cells were grown in the basal medium with 1 ng·mL^−1^ of EGF compared with those cultured in basal media with the other single component (Fig. 1A, left). We also observed a significant decrease in MMS in cells cultured in ‘complete’ EpiLife^®^ without EGF compared with ‘complete’ EpiLife^®^ media lacking a single component (Fig. 1A, right). Furthermore, we also found that PDT significantly decreased when cells were grown in basal medium with 1 ng·mL^−1^ of EGF relative to those cultured in basal media with the other single component (Fig. 1B, left). However, we did not observe significant changes in PDT among cells cultured in ‘complete’ medium and in any of the media lacking an individual component (Fig. 1B, right). Taken together, these results suggest that the presence of EGF in the growth supplement is likely to play a key role in regulating cell motility and proliferation.

### Effect of siRNA treatment on the cell motility of cells cultured in ‘complete’ EpiLife ^®^

**Fig. 2:**
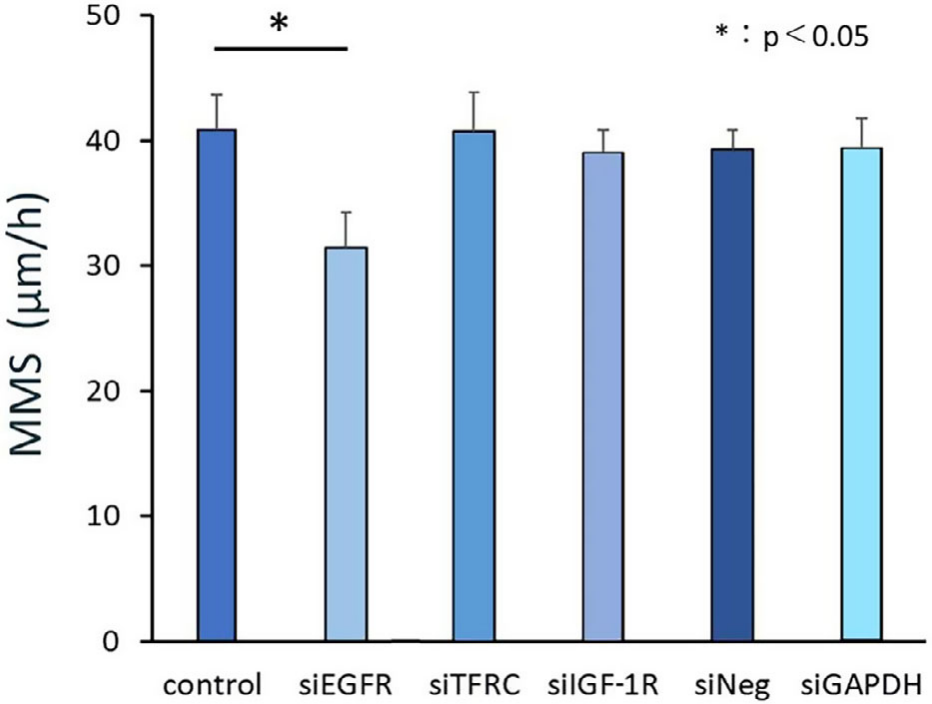
Effect of siRNA treatment on cell motility of cells cultured in ‘complete’ EpiLife®. Mean MMS (mean motion speed: an index of cell motility: *N* = 3) values for oral keratinocytes cultured in a complete medium were significantly reduced by EGFR‐knockdown. Data are shown as the mean ± SD. Significant differences among the groups were determined by one‐way ANOVA with Tukey's *post hoc* tests. *P < 0.05.

Next, we analyzed the effect of EGFR, TFRC, and IGF‐1R siRNA on the cell motility of oral keratinocytes. We found that the MMS of cells treated with EGFR siRNA decreased significantly relative to cells treated with TFRC, IGF‐1R, and GAPDH siRNA, as well as relative to a negative control siRNA treatment or a lipofectamine treatment (Fig. 2). These results suggest that the EGF/EGFR ligand system can stimulate signaling pathways that mediate cell motility and proliferation.

### The effect of pharmacological inhibition of EGFR on oral keratinocyte cell motility and proliferation

**Fig. 3:**
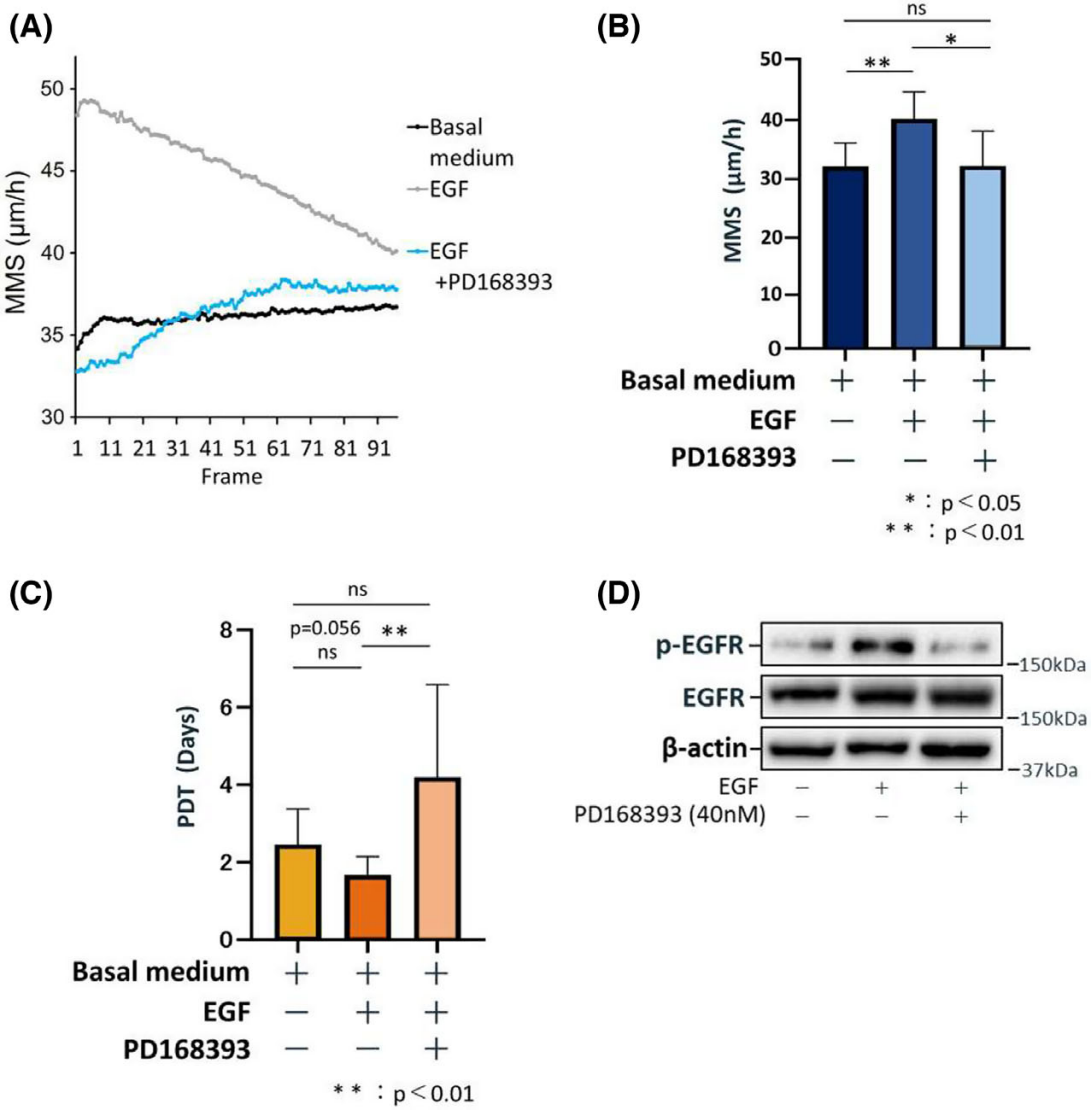
PD168393, a specific EGFR inhibitor, affects oral keratinocyte cell motility and proliferative capacity. (A) Change in MMS (mean motion speed: an index of cell motility: *N* = 10) over time (24 h) measured in 96 frames. Cells were cultured in a basal medium without EGF, a basal medium containing 1 ng·mL^−1^ EGF, and a basal medium containing 1 ng·mL^−1^ EGF and 40 nm PD168393. (B) Mean MMS values for all 96 frames are shown to compare cells cultured in a basal medium without EGF, a basal medium containing 1 ng·mL^−1^ EGF, and a basal medium containing 1 ng·mL^−1^ EGF and 40 nm PD168393. Data are shown as the mean ± SD. Significant differences among the groups were determined by one‐way ANOVA with Tukey's *post hoc* tests. *P < 0.05, and **P < 0.01. (C) Mean PDT (population doubling time: an index of proliferative capacity: *N* = 10) values are shown to compare cells cultured in a basal medium without EGF, a basal medium containing 1 ng·mL^−1^ EGF, and a basal medium containing 1 ng·mL^−1^ EGF and 40 nm PD168393. Data are shown as the mean ± SD. Significant differences among the groups were determined by one‐way ANOVA with Tukey's *post hoc* tests. **P < 0.01. (D) Representative immunoblot images for the proteins involved in EGFR signaling. Cells were cultured in a basal medium without EGF, a basal medium containing 1 ng·mL^−1^ EGF, and a basal medium containing 1 ng·mL^−1^ EGF and 40 nm PD168393.

To further confirm the putative role of EGFR‐mediated signal transduction, we examined the effect of a specific EGFR inhibitor, PD168393 [22], on oral keratinocyte cell motility and proliferation. As a negative control, we recorded the MMS of cells in basal medium alone; they showed a continuously low and unchanging MMS over 24 h (Video S1). As a positive control, we recorded the MMS of cells cultured in basal medium containing EGF; they showed MMS values that were never lower than that of the negative control, but declined continuously over the course of the experiment (Video S2). Data for these controls are shown in Fig. 3A. As expected, PD168393 was associated with a remarkable decrease in the MMS of the cells (Fig. 3A; Video S3), and this effect was strong enough to significantly diminish the average MMS value over the course of time‐lapse data acquisition under the microscope (Fig. 3B). In addition, PD168393 significantly increased PDT (Fig. 3C) and immunoblotting also identified a reduction in p‐EGFR (Tyr1092; Fig. 3D). Taken together, these results suggest that the EGF/EGFR signal transduction pathway plays a major role in oral keratinocyte cell and colony motility and proliferative capacity.

### EGFR‐mediated downstream cascades regulate oral keratinocyte cell motility and proliferation

**Fig. 4:**
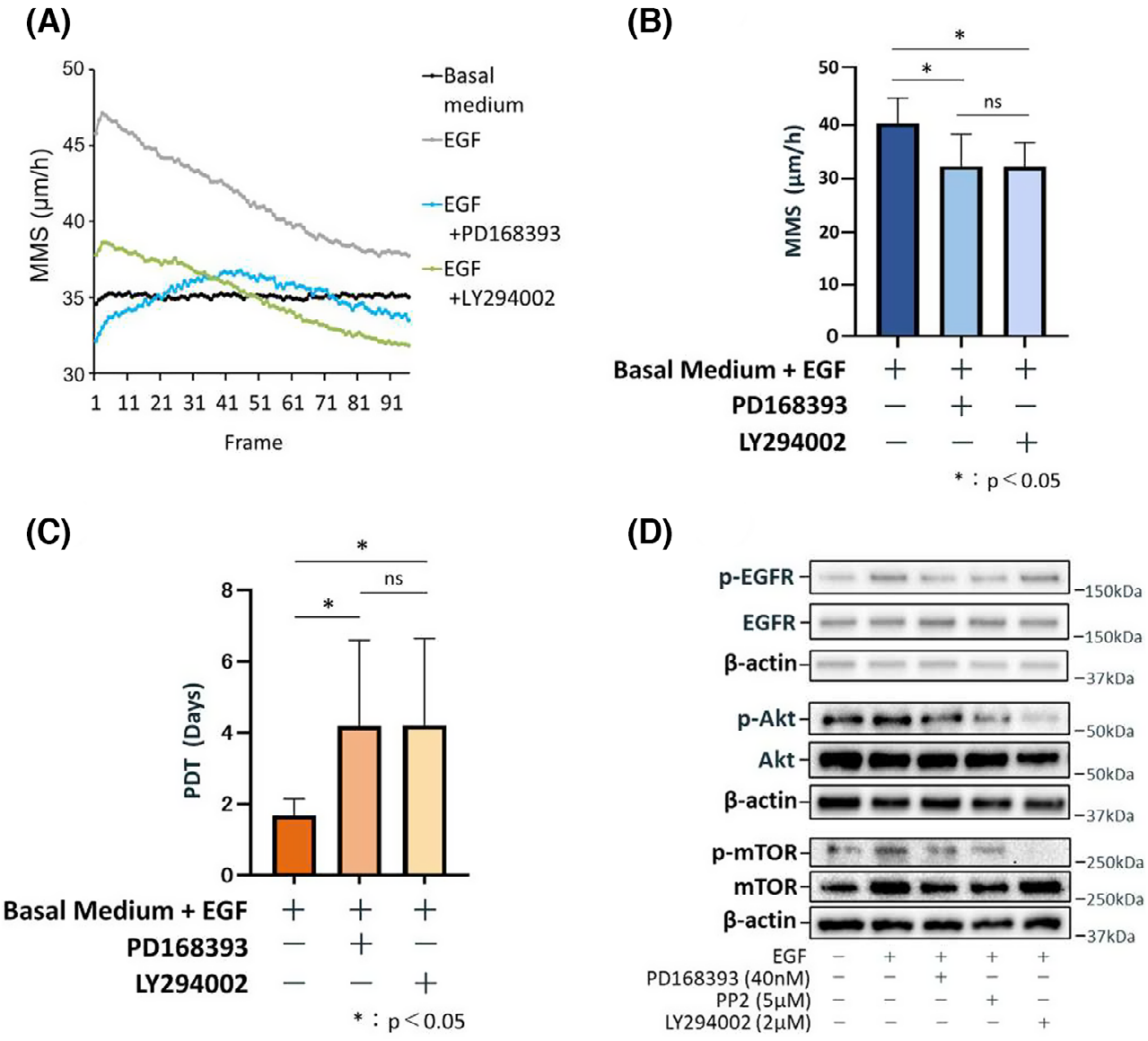
LY294002, a specific PI3K/Akt inhibitor, affects oral keratinocyte cell motility and proliferative capacity. (A) Change in MMS (mean motion speed: an index of cell motility: *N* = 10) over time (24 h) measured in 96 frames. Cells were cultured in a basal medium without EGF, a basal medium containing 1 ng·mL^−1^ EGF, and a basal medium containing 1 ng·mL^−1^ EGF and 40 nm PD168393 or 2 μm LY294002. (B) Mean MMS values for all 96 frames are shown to compare among cells cultured in a basal medium containing 1 ng·mL^−1^ EGF and a basal medium containing 1 ng·mL^−1^ EGF and 40 nm PD168393 or 2 μm LY294002. Data are shown as the mean ± SD. Significant differences among the groups were determined by one‐way ANOVA with Tukey's *post hoc* tests. *P < 0.05. (C) Mean PDT (population doubling time: an index of proliferative capacity: *N* = 10) values are shown to compare cells cultured in a basal medium containing 1 ng·mL^−1^ EGF and a basal medium containing 1 ng·mL^−1^ EGF and 40 nm PD168393 or 2 μm LY294002. Data are shown as the mean ± SD. Significant differences among the groups were determined by one‐way ANOVA with Tukey's *post hoc* tests. *P < 0.05. (D) Representative immunoblot images for proteins involved in EGFR/Akt/mTOR signaling are shown. Cells were cultured in a basal medium containing 1 ng·mL^−1^ EGF and a basal medium containing 1 ng·mL^−1^ EGF and 40 nm PD168393, 5 μm PP2 (an inhibitor of Src family kinases), or 2 μm LY294002.

Next, we examined the downstream signaling cascades mediated by the EGF/EGFR axis. We found that the addition of LY294002, a PI3K/Akt inhibitor, significantly decreased the MMS of oral keratinocytes cultured in basal medium containing EGF (Video S4), and this effect was similar to that of PD168393 (Fig. 4A,B). Moreover, LY294002 also significantly increased PDT (Fig. 4C). Immunoblotting demonstrated that LY294002 diminished the expression levels of p‐Akt (Ser473) and p‐mTOR (Ser2448; Fig. 4D), which were both also slightly downregulated by PD168393 and PP2, a Src inhibitor, (Fig. 4D). However, LY294002 did not block p‐EGFR (Tyr1092).

**Fig. 5:**
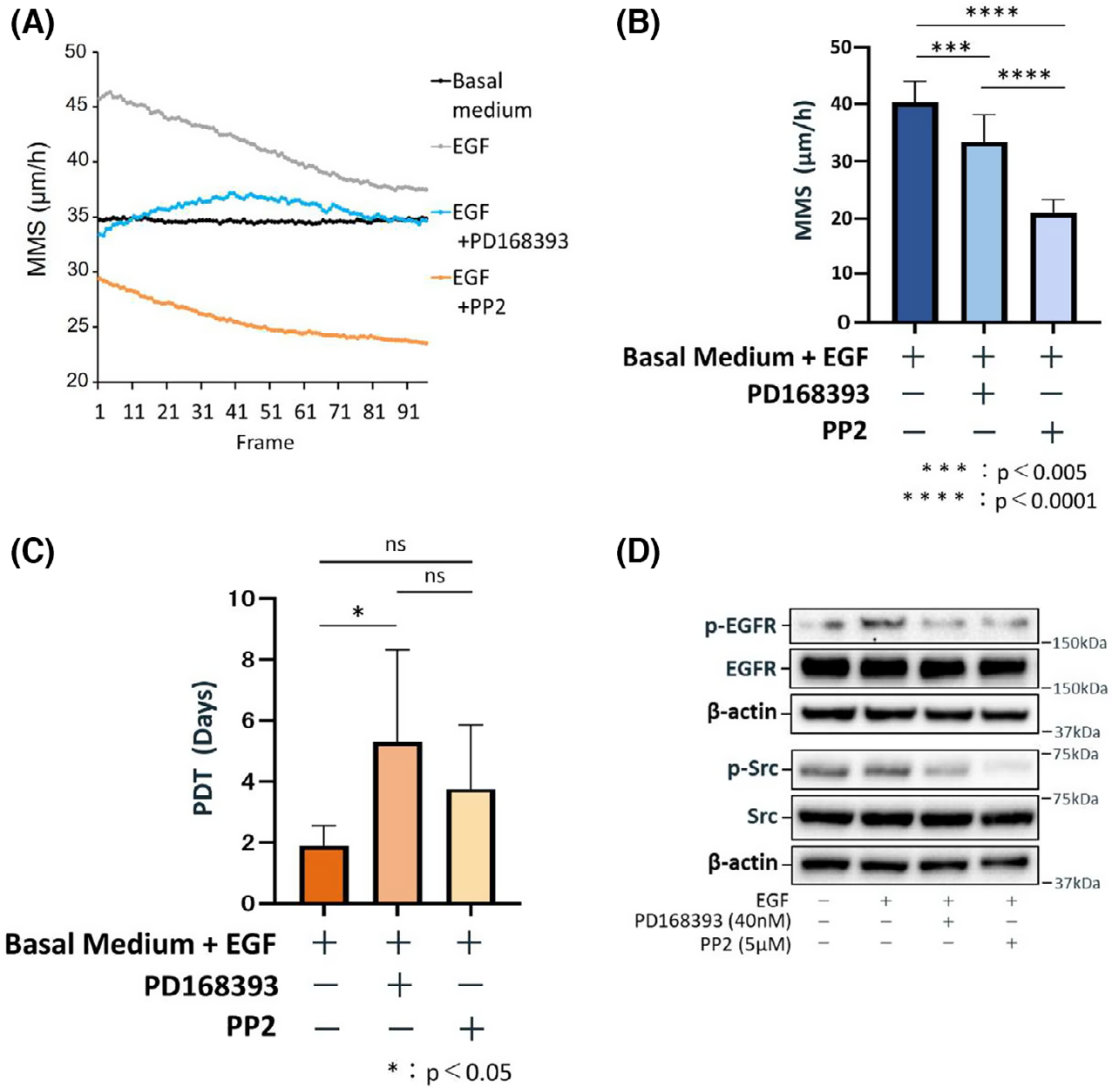
PP2, a specific Src family kinase inhibitor, affects oral keratinocyte cell motility and proliferative capacity. (A) Change in MMS (mean motion speed: an index of cell motility: *N* = 10) over time (24 h) measured in 96 frames. Cells were cultured in a basal medium without EGF, a basal medium containing 1 ng·mL^−1^ EGF, and a basal medium containing 1 ng·mL^−1^ EGF and 40 nm PD168393 or 5 μm PP2. (B) Mean MMS values for all 96 frames are shown to compare among cells cultured in a basal medium containing 1 ng·mL^−1^ EGF and a basal medium containing 1 ng·mL^−1^ EGF and 40 nm PD168393 or 5 μm PP2. Data are shown as the mean ± SD. Significant differences among the groups were determined by one‐way ANOVA with Tukey's *post hoc* tests. ***P < 0.005, and ****P < 0.0001. (C) The averages of PDT (population doubling time: an index of proliferative capacity: *N* = 10) are shown to compare cells cultured in a basal medium containing 1 ng·mL^−1^ EGF and a basal medium containing 1 ng·mL^−1^ EGF and 40 nm PD168393 or 5 μm PP2. Data are shown as the mean ± SD. Significant differences among the groups were determined by one‐way ANOVA with Tukey's *post hoc* tests. *P < 0.05. (D) Representative immunoblot images for proteins involved in EGFR/Src signaling are shown. Cells were cultured in a basal medium containing 1 ng·mL^−1^ EGF and a basal medium containing 1 ng·mL^−1^ EGF and 40 nm PD168393 or 5 μm PP2.

PP2 was also associated with reduced MMS in oral keratinocytes cultured in basal medium containing EGF (Video S5). However, for this treatment, MMS continued to decline over the course of the experiment, reaching values that were remarkably lower than those following PD168393 treatment (Fig. 5A,B). Although PP2 moderately increased PDT, its inhibitory effect on proliferative capacity was not statistically significant; this was also different from PD168393 (Fig. 5C). Immunoblotting confirmed that PD168393 and PP2 both reduced the expression of p‐EGFR (Fig. 5D). Finally, p‐Src levels were found to be blocked by both PP2 and PD168393.

In contrast to PD168393 and PP2, LLL12, a STAT3 phosphorylation inhibitor, showed a minimal, non‐statistically significant effect on the MMS of oral keratinocytes cultured in basal medium containing EGF (Fig. S1A,B; Video S6). Moreover, LLL12 increased the PDT but did not make statistical differences, which indicates that it shows a mild inhibitory effect on the proliferative capacity of oral keratinocytes (Fig. S1C). Finally, immunoblotting showed that PD168393, PP2, and LLL12 reduced the level of p‐STAT3 although PD168393 did not to the same degree as PP2 and LLL12 (Fig. S1D).

Next, we found that U0126, a MEK1/2 inhibitor, slightly increased the MMS of oral keratinocytes cultured in basal medium containing EGF (Fig. S2A,B; Video S7), which is in contrast to the effect of PD168393. In addition, PDT values treated with U0126 were unaltered and similar to that of the basal medium containing EGF, indicating that it does not suppress the proliferative capacity of oral keratinocytes (Fig. S2C). Immunoblotting demonstrated that PD168393, PP2, and U0126 suppressed the expression of p‐ERK1/2(MAPK), while PP2 did not to the same degree as PD168393 and U0126 (Fig. S2D).

The MMS and PDT of oral keratinocytes cultured in basal medium containing EGF treated with PF‐562271, a FAK inhibitor, did not differ from cells cultured without this additive (Fig. S3A–C). Thus, we conclude that it shows little suppressive effect on oral keratinocyte cell motility and proliferative capacity. Immunoblotting showed that PF‐562271 suppressed the expression of p‐FAK while PD168393, LY294002, and PP2 did not (Fig. S3D).

**Fig. 6:**
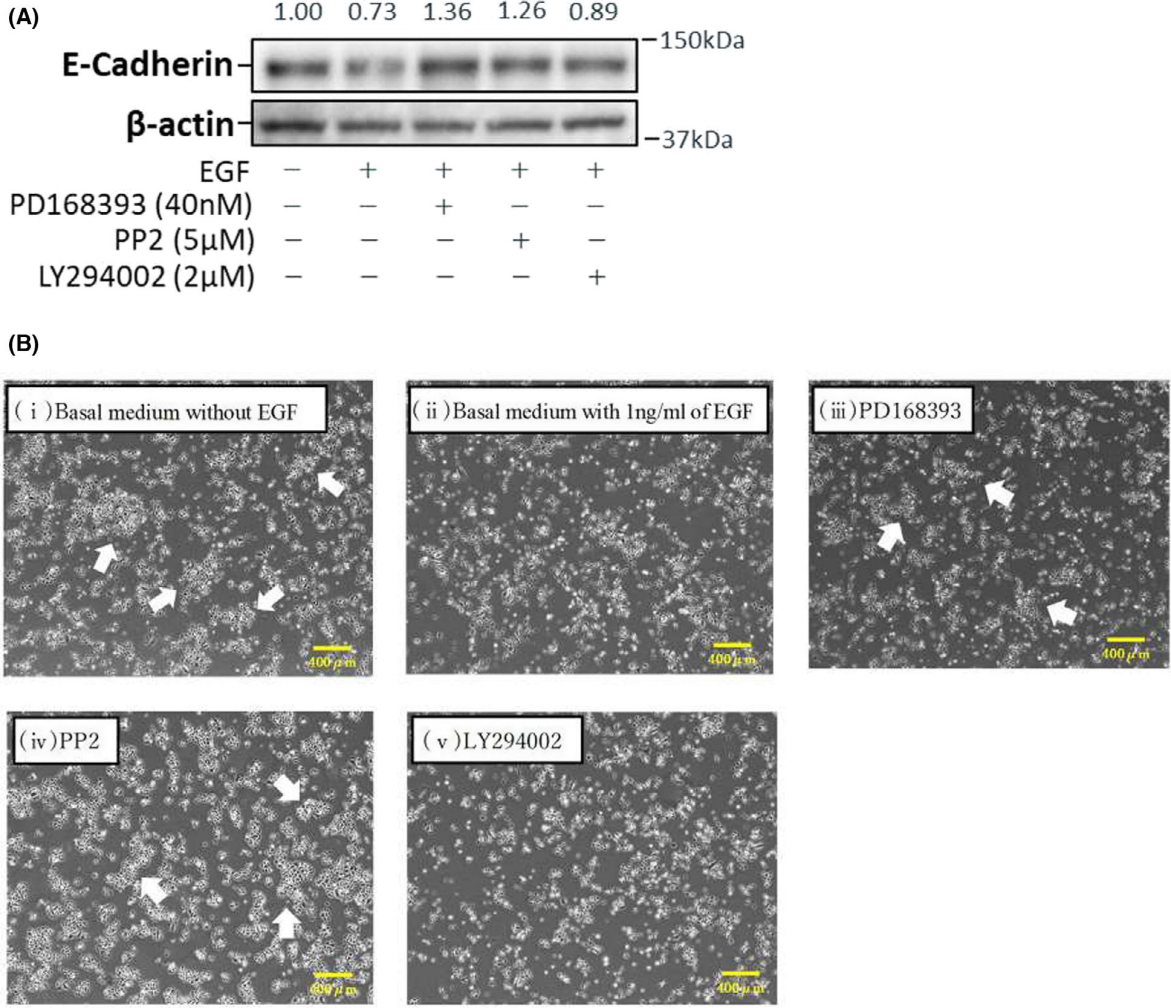
E‐cadherin expression levels of oral keratinocytes cultured in the basal medium with and without treatment with specific pharmacological inhibitors of EGFR, Src, and PI3K/Akt. (A) Representative immunoblot images are shown (*N* = 5). The intensity of the E‐cadherin band from representative gels was quantified and normalized to β‐actin. Densitometry values represent the expression ratio of the treatment versus the control (i.e., the expression level of cells cultured in the basal medium without EGF). (B) Growth pattern of oral keratinocytes. Cells treated with 5 μm PP2 and 40 nm PD168393, as well as those cultured in the basal medium, favored colony formation (white arrows). In contrast, cells treated with 2 μm LY294002 and those cultured in the basal medium with EGF were scattered. Representative phase‐contrast microscopic images of oral keratinocytes grown in the culture medium with and without specific inhibitors. (i) Basal medium without EGF, (ii) Basal medium with 1 ng·mL^−1^ of EGF, (iii) 40 nm PD168393, (iv) 5 μm PP2, and (v) 2 μm LY294002. Cells were photographed using a ×1.6 objective. Scale bar = 400 μm.

Finally, we also examined the expression level of E‐cadherin following treatment with inhibitors because we observed significant reductions of MMS in the PD168393, LY294002, and PP2 treatments (Fig. 6A). Compared with oral keratinocytes cultured in basal medium containing EGF, E‐cadherin expression was enhanced in cells treated with PD168393 and PP2 but was unaltered in those treated with LY294002 (Fig. 6A). This finding was consistent with the growth patterns observed in the PD168393 and PP2 treatments, in which colony formation was favored, in contrast to the pattern observed in the LY294002 treatment, which favored cell scattering (Fig. 6B).

## Discussion

To date, our understanding of which signals regulate or are otherwise involved in oral keratinocyte cell motility and proliferative capacity remains poor. However, a recent report stated that EGF reversed antimotogenic and mitogenic effects of bisphosphonates via the EGFR/PI3K/Akt signaling pathway [23]. To the best of our knowledge, this study is the first to demonstrate that the EGF/EGFR axis and its downstream signaling cascade of Src/PI3K/Akt/mTOR (i.e., rather than the STAT3 and ERK1/2 pathways) is confirmed to play a major role in regulating cell motility and proliferative capacity during oral keratinocyte cell expansion. Moreover, this finding is consistent with previous studies of corneal and skin keratinocytes [24, 25]. Our findings indicate that oral keratinocytes can maintain motility and proliferative capacity via EGF/EGFR‐mediated signal transduction in a ‘complete’ medium, which provides new insight into the QC of oral keratinocytes for therapeutic use [26]. We also found that the STAT3 and ERK1/2 pathways, two other major EGFR downstream cascades, showed minor effects on oral keratinocyte cell motility and proliferation although PD168393, a specific pharmacological inhibitor of EGFR, downregulated ERK1/2 (MAPK) more than STAT3 and PP2, Src specific inhibitor, downregulated STAT3 more than ERK1/2 (MAPK). However, this finding was not consistent with previous studies, which reported that both pathways enhanced motogenic and mitogenic activity in normal epithelial cells [27, 28]. Since EGFR downstream cascades interconnect, engage in cross‐talk, and can be dependent on multiple levels, exactly how the Src/PI3K/Akt/mTOR, STAT3, and ERK1/2 signal pathways interact in oral keratinocytes needs to be investigated further.

Many studies have shown that EGFR ligands activate PI3K/Akt/mTOR signaling and promote motogenic and/or mitogenic effects on skin keratinocytes [29, 30, 31], and many reagents capable of stimulating PI3K/Akt/mTOR signaling are available. Therefore, our finding may be beneficial for pharmacological manipulation during oral keratinocyte cell expansion, an initial phase of the formation of the oral mucosa epithelial cell sheet [13, 26]. Nonetheless, because the PI3K/Akt/mTOR signaling pathway has other critical cellular functions that may cause adverse effects, rigorous screening and evaluation of cell signaling modifiers are necessary prior to clinical application. One study showed that the migration of skin keratinocytes was regulated independently by both an EGF‐mediated MAPK pathway and an IGF‐1‐mediated PI3K pathway [32]. Although this discrepancy may be due to intrinsic characteristics of skin keratinocytes, further dissection of these pathways, both of which are coupled to a complex signal transduction network, can provide deeper insight into the different mechanisms involved in the migration of skin and oral keratinocytes.

The significant effect of the Src inhibitor PP2 on cell motility rather than proliferation shown in this study was consistent with a previous study of skin and gastric epithelial cells [33, 34]. The activity of Src, a non‐receptor tyrosine kinase and namesake member of the Src family kinases (SFKs), can transduce signals from various receptors to internal signaling pathways. This affects diverse cellular events including survival, adhesion, proliferation, and migration [35]. In addition, the mutual downregulation of p‐EGFR and p‐Src by PD168393 and PP2, respectively, demonstrates that there is a reciprocal interaction between Src and EGFR in oral keratinocytes. Moreover, this also suggests an upstream signal transduction from Src to the EGF/EGFR axis in oral keratinocytes. Such transduction would be consistent with a previous study of the corneal epithelium which showed that SFKs mediate EGFR transactivation [36]. Therefore, EGFR and downstream Src/PI3K/Akt/mTOR activation may also be dependent on Src activation. Nonetheless, Src also interacts with many other intracellular molecules that facilitate cell motility and proliferation, including FAK, which is activated by integrin stimulation. Further studies are required to clarify whether the effects explored here reflect interactions between Src and integrin‐mediated FAK [35, 37, 38].

**Fig. 7:**
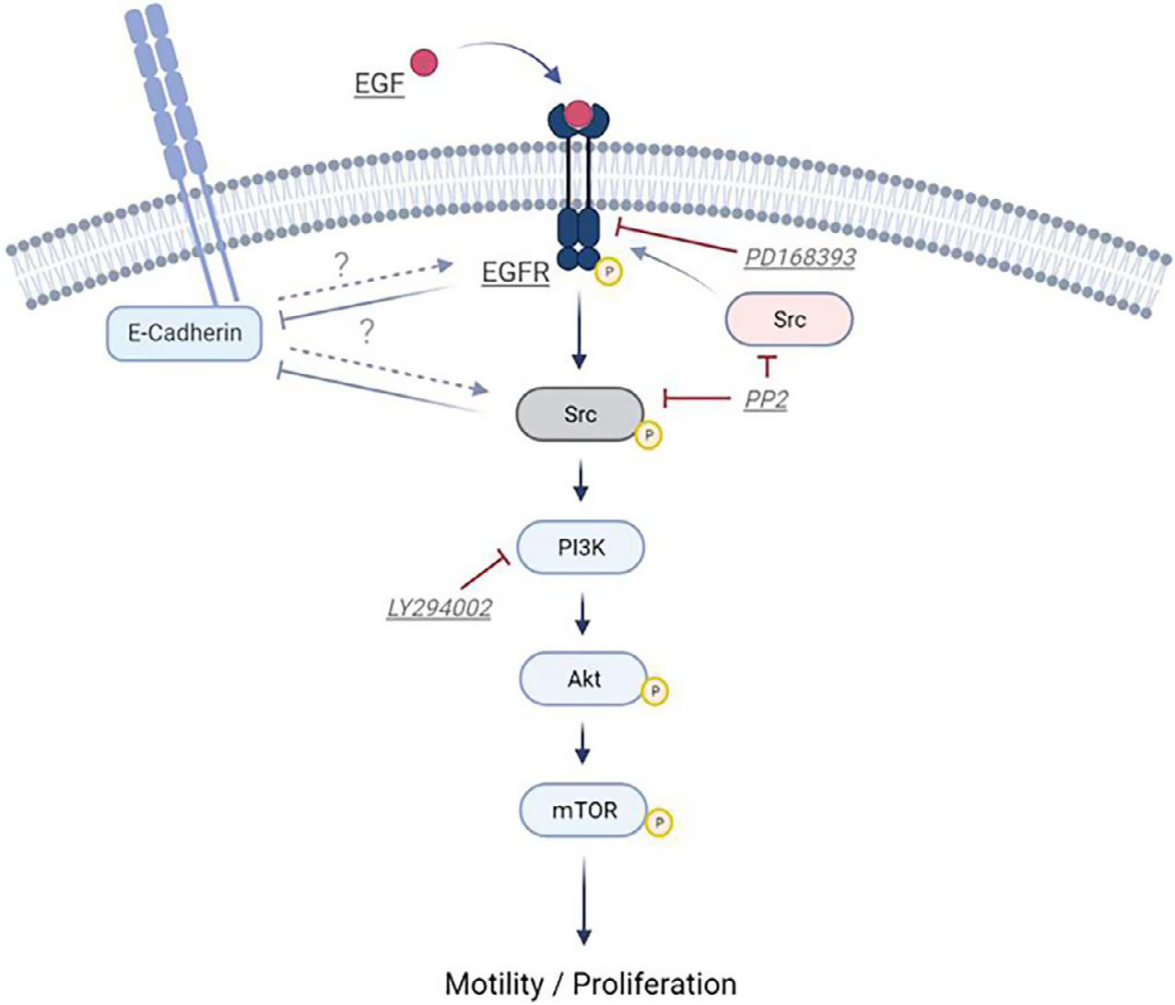
Graphic illustration of the EGF/EGFR‐mediated signaling pathways of oral keratinocytes. Under the culture conditions of this study, activation of the EGFR/Src/PI3K/Akt/mTOR signaling pathway results in the enhancement of cell proliferation and in the migration of oral keratinocytes. Scheme created with BioRender.com.

Studies of skin keratinocytes have demonstrated that there is substantial cross‐talk between EGFR and E‐cadherin [39, 40]. Increased Src/EGFR signaling is correlated with decreased E‐cadherin expression, which results in enhanced cell migration [41]. In the presence of PD168393 and PP2, which inhibit the activation of EGFR, the expression levels of E‐cadherin were elevated. This elevation is associated with a characteristic growth pattern in which cells favor colony formation. These findings are consistent with a significant reduction in cells/colony motion because E‐cadherin is an adhesion molecule that senses the extracellular environment, including cell–cell attachment and the extracellular matrix. Taken together, these findings suggest that EGFR and Src negatively regulate the expression of E‐cadherin. Nonetheless, we also found that LY294002 did not upregulate the expression of E‐cadherin, which is consistent with a growth pattern based on cell scattering. However, it could also be attributed to p‐EGFR downregulation and a significant reduction of MMS. This inconsistency might be caused by little cross‐talk occurring between E‐cadherin and EGFR downstream signal transduction in oral keratinocytes. Further research is necessary to elucidate how EGFR signal transduction is involved in cell adhesion, which is also involved in regulation of cell motility and proliferation, since E‐cadherin‐mediated cell attachment plays an important role in the development of epithelial cell sheets associated with collective cell motion [12, 42, 43, 44]. Figure 7 shows a schematic illustration of the EGF/EGFR‐mediated signaling pathways in oral keratinocytes as revealed by this study.

We note that a relatively low concentration of EGF (1 ng·mL^−1^) was supplemented in our culture system, whereas the EGF concentrations used in previous studies varied [45, 46]. However, one study reported that the phosphorylation of p130^Cas^, one of the EGFR/Src downstream molecules, exhibited a bell‐shaped dose–response curve following EGF stimulation [47]. Moreover, another recent study of skin wound healing using 10 ng·mL^−1^ of EGF showed that EGF/EGFR signaling declined with age, and this also influenced keratinocyte clonal growth [12, 48]. Although further research is necessary to elucidate the regulatory mechanisms responsible for these changes, attention should be paid to the fact that differential cellular responses appear to be dependent on EGF concentration in *in vitro* studies.

Amplification and/or maintenance of cultured keratinocyte stem cells during *ex vivo* cell expansion and subsequent cell sheet formation is a prerequisite for the successful clinical translation of the epithelial cell sheet [49]. Nonetheless, to date, no oral mucosa keratinocyte stem cell markers have been identified [9]. Even if identified, most of the methodologies for examining molecular marker expression are invasive and require the destruction of cells, which is an unfavorable form of QC for tissue‐engineering products. Therefore, cell motility as indexed by MMS can be used as an alternative marker of oral keratinocyte quality during oral keratinocyte cell expansion, since it is strongly correlated with proliferative capacity [11, 16]. Although high cell quality is necessary for consistent, large‐scale biomanufacturing [50], the *in vitro* culture environment does not appear to manifest ideal conditions for the maintenance of keratinocyte stem cell phenotypes [10]. Recently, pharmacological approaches using cell signaling modifiers can facilitate keratinocyte progenitor/stem cell expansion and/or maintenance. This, in turn, makes available a wider array of strategies for their therapeutic use and minimizes the initial variation and heterogeneity of primary oral keratinocyte populations [27, 51, 52]. The logistics of producing a large‐scale and efficient expansion of cultured oral keratinocytes (compared with the size of autografts) from a small tissue sample is a vital issue for regenerative medicine. Thus, the findings of this study provide new insight into potential pharmacological approaches using reagents that activate the EGFR/Src [26, 29, 53], for the efficient isolation, expansion, and sheet formation of cultured oral keratinocytes. However, pharmacological‐based manipulation through the induction of the activation of EGFR/Src has to be proceeded with caution because activation of the EGFR/Src signaling pathway could stimulate oncogenic signals [54, 55].

In conclusion, this work demonstrated that EGF is a key component in the oral keratinocyte culture system used in this study, and that the EGF/EGFR axis and its downstream Src/PI3K/Akt/mTOR signaling cascade plays a major role in regulating oral keratinocyte cell motility and proliferative capacity. This finding was also supported by the effect of specific inhibitors of EGFR and Src that was correlated with E‐cadherin expression and the growth patterns of cultured oral keratinocytes. These findings suggest possible pharmacological approaches that will use beneficial signaling modifiers to enable the QC of oral keratinocytes for therapeutic use.

## Conflict of interest

The authors declare no conflict of interest.

### Peer review

The peer review history for this article is available at https://www.webofscience.com/api/gateway/wos/peer‐review/10.1002/2211‐5463.13653.

## Author contributions

RK, EH, SI, HH, and KI conceived and designed the work. RK, EH, TS, OS, EN, AS, and KI conducted the experiments. RK, EH, TS, OS, EN, AS, SI, HH, and KI analyzed the data. RK, TS, KT, and KI contributed reagents/materials/analysis tools. RK, EH, TS, and KI visualized data. RK and KI wrote the original draft of the paper. SI, HH, KT, and KI edited and reviewed the paper. All authors have read and agreed to the published version of the manuscript.

## Supporting information


**Fig. S1.** Effects of LLL12, a specific STAT3 phosphorylation inhibitor, on the cell motility and proliferative capacity of oral keratinocytes. (A) Changes in the MMS (mean motion speed, an index of cell motility: *N* = 10) over a period of 24 h consisting of 96 frames. Cells were cultured in a basal medium without EGF, a basal medium containing 1 ng·mL^−1^ EGF, and a basal medium containing 1 ng·mL^−1^ EGF and either 40 nm PD168393 or 200 nm LLL12. (B) The mean values of MMS for all 96 frames are shown to compare the motility of cells cultured in a basal medium containing 1 ng·mL^−1^ EGF and a basal medium containing 1 ng·mL^−1^ EGF and either 40 nm PD168393 or 200 nm LLL12. Data are shown as the mean ± SD. Significant differences among the groups were determined by one‐way ANOVA with Tukey's *post hoc* tests. *P < 0.05. (C) Mean values of PDT (population doubling time, an index of proliferative capacity: *N* = 10) are shown to compare the PDT of cells cultured in a basal medium containing 1 ng·mL^−1^ EGF and a basal medium containing 1 ng·mL^−1^ EGF and either 40 nm PD168393 or 200 nm LLL12. Data are shown as the mean ± SD. (D) Representative immunoblot images for proteins involved in STAT3 signaling are shown. Cells were cultured in a basal medium containing 1 ng·mL^−1^ EGF and a basal medium containing 1 ng·mL^−1^ EGF and either 40 nm PD168393, 5 μm PP2, or 200 nm LLL12.

**Fig. S2.** U0126, a specific MEK1/2 inhibitor, affects oral keratinocyte cell motility and proliferative capacity. (A) Changes in the MMS (mean motion speed, an index of cell motility: *N* = 10) over a period of 24 h consisting of 96 frames. Cells were cultured in a basal medium without EGF, a basal medium containing 1 ng·mL^−1^ EGF, and a basal medium containing 1 ng·mL^−1^ EGF and either 40 nm PD168393 or 200 nm U0126. (B) The mean values of MMS for all 96 frames are shown to compare the motility of cells cultured in a basal medium containing 1 ng·mL^−1^ EGF and a basal medium containing 1 ng·mL^−1^ EGF and either 40 nm PD168393 or 200 nm U0126. Data are shown as the mean ± SD. Significant differences among the groups were determined by one‐way ANOVA with Tukey's *post hoc* tests. ***P < 0.005, and ****P < 0.0001. (C) Mean values of PDT (population doubling time, an index of proliferative capacity: *N* = 10) are shown to compare the PDT of cells cultured in a basal medium containing 1 ng·mL^−1^ EGF and a basal medium containing 1 ng·mL^−1^ EGF and either 40 nm PD168393 or 200 nm U0126. Data are shown as the mean ± SD. Significant differences among the groups were determined by one‐way ANOVA with Tukey's *post hoc* tests. *P < 0.05. (D) Representative immunoblot images for proteins involved in MAPK signaling are shown. Cells were cultured in a basal medium containing 1 ng·mL^−1^ EGF and a basal medium containing 1 ng·mL^−1^ EGF and either 40 nm PD168393, 5 μm PP2, or 200 nm U0126.

**Fig. S3.** PF‐562271, a specific FAK inhibitor, affects oral keratinocyte cell motility and proliferative capacity. (A) Changes in the MMS (mean motion speed: an index of cell motility: *N* = 10) over a period of 24 h consisting of 96 frames. Cells were cultured in a basal medium without EGF, a basal medium containing 1 ng·mL^−1^ EGF, and a basal medium containing 1 ng·mL^−1^ of EGF and either 40 nm PD168393 or 3 μm PF‐562271. (B) The mean values of MMS for all 96 frames are shown to compare the motility of cells cultured in a basal medium with 1 ng·mL^−1^ of EGF and a basal medium containing 1 ng·mL^−1^ of EGF and either 40 nm PD168393 or 3 μm PF‐562271. Data are shown as the mean ± SD. Significant differences among the groups were determined by one‐way ANOVA with Tukey's *post hoc* tests. *P < 0.05. (C) Mean values of PDT (population doubling time: an index of proliferative capacity: *N* = 10) are shown to compare the PDT of cells cultured in a basal medium with 1 ng·mL^−1^ EGF and a basal medium containing 1 ng·mL^−1^ of EGF and either 40 nm PD168393 or 3 μm F‐562271. Data are shown as the mean ± SD. Significant differences among the groups were determined by one‐way ANOVA with Tukey's *post hoc* tests. *P < 0.05. (D) Representative immunoblot images for proteins involved in FAK signaling are shown. Cells were cultured in a basal medium containing 1 ng·mL^−1^ of EGF and a basal medium containing 1 ng·mL^−1^ of EGF and either 40 nm PD168393, 2 μm LY294002, 5 μm PP2, or 3 μm PF‐562271.

**Video S1.** Cells were cultured in the basal medium without EGF.

**Video S2.** Cells were cultured in the basal medium containing 1 ng·mL^−1^ EGF.

**Video S3.** Cells were cultured in the basal medium containing 1 ng·mL^−1^ EGF and 40 nm PD168393, a specific EGFR inhibitor.

**Video S4.** Cells were cultured in the basal medium containing 1 ng·mL^−1^ EGF and 2 μm LY294002, a specific PI3K/Akt inhibitor.

**Video S5.** Cells were cultured in the basal medium containing 1 ng·mL^−1^ EGF and 5 μm PP2, a specific Src inhibitor.

**Video S6.** Cells were cultured in the basal medium containing 1 ng·mL^−1^ EGF and 200 nm LLL12, a specific STAT3 inhibitor.

**Video S7.** Cells were cultured in the basal medium containing 1 ng·mL^−1^ EGF and 200 nm U0126, a specific ERK1/2 inhibitor.

## Data Availability

The data that support the findings of this study are available from the corresponding author izumik@dent.niigata-u.ac.jp upon reasonable request.
