# Supplementary material for: The EGF/EGFR axis and its downstream signaling pathways regulate the motility and proliferation of cultured oral keratinocytes
Source: FEBS Open Bio. 2023 Jun 4;13(8):1469–84. doi: 10.1002/2211-5463.13653 (PMC10392064; doi:10.1002/2211-5463.13653)
Supplement: Supplementary file 1 — Fig. S1. Effects of LLL12, a specific STAT3 phosphorylation inhibitor, on the cell motility and proliferative capacity of oral keratinocytes. (A) Changes in the MMS (mean motion speed, an index of cell motility: N = 10) over a period of 24 h consisting of 96 frames. Cells were cultured in a basal medium without EGF, a basal medium containing 1 ng·mL−1 EGF, and a basal medium containing 1 ng·mL−1 EGF and either 40 nm PD168393 or 200 nm LLL12. (B) The mean values of MMS for all 96 frames are shown to compare the motility of cells cultured in a basal medium containing 1 ng·mL−1 EGF and a basal medium containing 1 ng·mL−1 EGF and either 40 nm PD168393 or 200 nm LLL12. Data are shown as the mean ± SD. Significant differences among the groups were determined by one‐way ANOVA with Tukey's post hoc tests. *P < 0.05. (C) Mean values of PDT (population doubling time, an index of proliferative capacity: N = 10) are shown to compare the PDT of cells cultured in a basal medium containing 1 ng·mL−1 EGF and a basal medium containing 1 ng·mL−1 EGF and either 40 nm PD168393 or 200 nm LLL12. Data are shown as the mean ± SD. (D) Representative immunoblot images for proteins involved in STAT3 signaling are shown. Cells were cultured in a basal medium containing 1 ng·mL−1 EGF and a basal medium containing 1 ng·mL−1 EGF and either 40 nm PD168393, 5 μm PP2, or 200 nm LLL12. [file FEB4-13-1469-s001.pdf]

Supplementary Figure 1

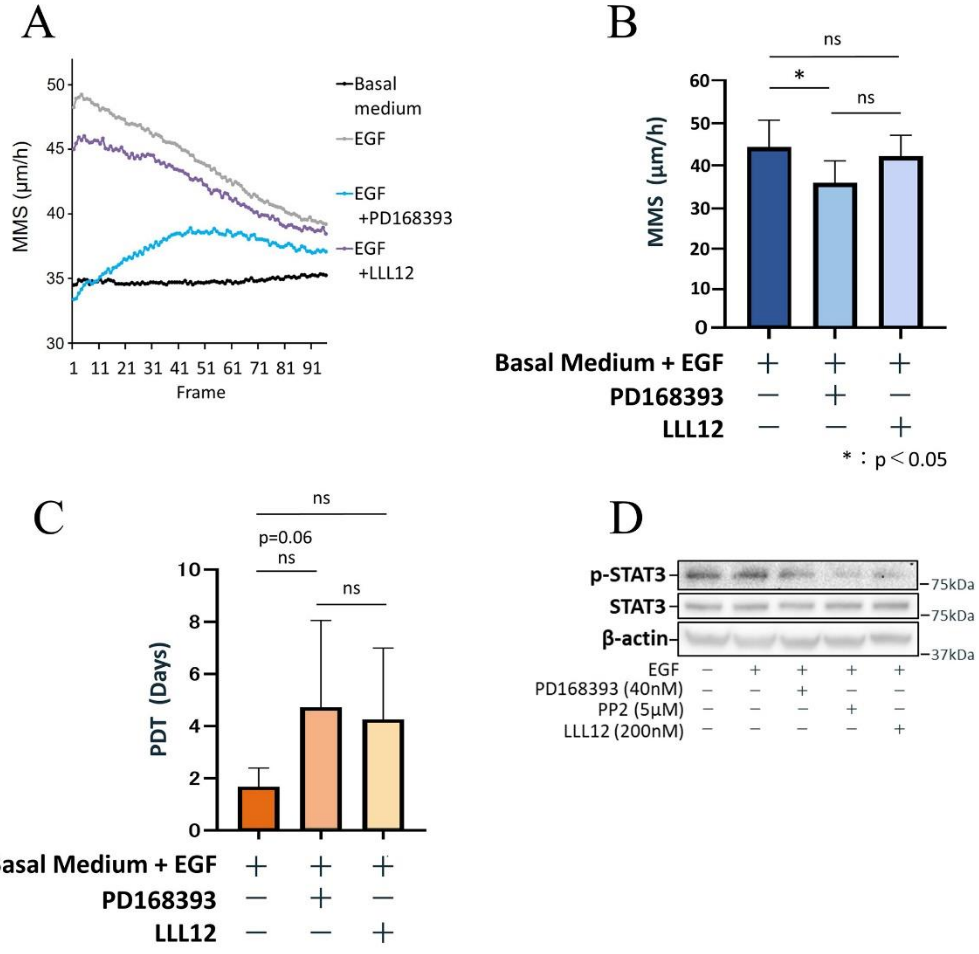

Supplementary Figure 1: The effects of LLL12, a specific STAT3 phosphorylation inhibitor, on the cell motility and proliferative capacity of oral keratinocytes.

(A) Changes in the MMS (mean motion speed, an index of cell motility: N = 10) over a period of 24 h consisting of 96 frames. Cells were cultured in a basal medium without EGF, a basal medium containing 1 ng/mL EGF, and a basal medium containing 1ng/mL EGF and either 40 nM PD168393 or 200 nM LLL12.

(B) The mean values of MMS for all 96 frames are shown to compare the motility of cells cultured in a basal medium containing 1 ng/mL EGF, and a basal medium containing 1 ng/mL EGF and either 40 nM PD168393 or 200 nM LLL12. Data are shown as the mean ± SD. Significant differences among the groups were determined by one-way analysis of variance with Tukey's post hoc tests. $*p < 0.05$.

(C) Mean values of PDT (population doubling time, an index of proliferative capacity: N = 10) are shown to compare the PDT of cells cultured in a basal medium containing 1 ng/mL EGF, and a basal medium containing 1 ng/mL EGF and either 40n M PD168393 or 200 nM LLL12. Data are shown as the mean ± SD.

(D) Representative immunoblot images for proteins involved in STAT3 signaling are shown. Cells were cultured in a basal medium containing 1 ng/mL EGF, and a basal medium containing 1 ng/mL EGF and either 40 nM PD168393, 5 μM PP2 or 200 nM LLL12.
