# Supplementary material for: The EGF/EGFR axis and its downstream signaling pathways regulate the motility and proliferation of cultured oral keratinocytes
Source: FEBS Open Bio. 2023 Jun 4;13(8):1469–84. doi: 10.1002/2211-5463.13653 (PMC10392064; doi:10.1002/2211-5463.13653)
Supplement: Supplementary file 2 — Fig. S2. U0126, a specific MEK1/2 inhibitor, affects oral keratinocyte cell motility and proliferative capacity. (A) Changes in the MMS (mean motion speed, an index of cell motility: N = 10) over a period of 24 h consisting of 96 frames. Cells were cultured in a basal medium without EGF, a basal medium containing 1 ng·mL−1 EGF, and a basal medium containing 1 ng·mL−1 EGF and either 40 nm PD168393 or 200 nm U0126. (B) The mean values of MMS for all 96 frames are shown to compare the motility of cells cultured in a basal medium containing 1 ng·mL−1 EGF and a basal medium containing 1 ng·mL−1 EGF and either 40 nm PD168393 or 200 nm U0126. Data are shown as the mean ± SD. Significant differences among the groups were determined by one‐way ANOVA with Tukey's post hoc tests. ***P < 0.005, and ****P < 0.0001. (C) Mean values of PDT (population doubling time, an index of proliferative capacity: N = 10) are shown to compare the PDT of cells cultured in a basal medium containing 1 ng·mL−1 EGF and a basal medium containing 1 ng·mL−1 EGF and either 40 nm PD168393 or 200 nm U0126. Data are shown as the mean ± SD. Significant differences among the groups were determined by one‐way ANOVA with Tukey's post hoc tests. *P < 0.05. (D) Representative immunoblot images for proteins involved in MAPK signaling are shown. Cells were cultured in a basal medium containing 1 ng·mL−1 EGF and a basal medium containing 1 ng·mL−1 EGF and either 40 nm PD168393, 5 μm PP2, or 200 nm U0126. [file FEB4-13-1469-s004.pdf]

Supplementary Figure 2

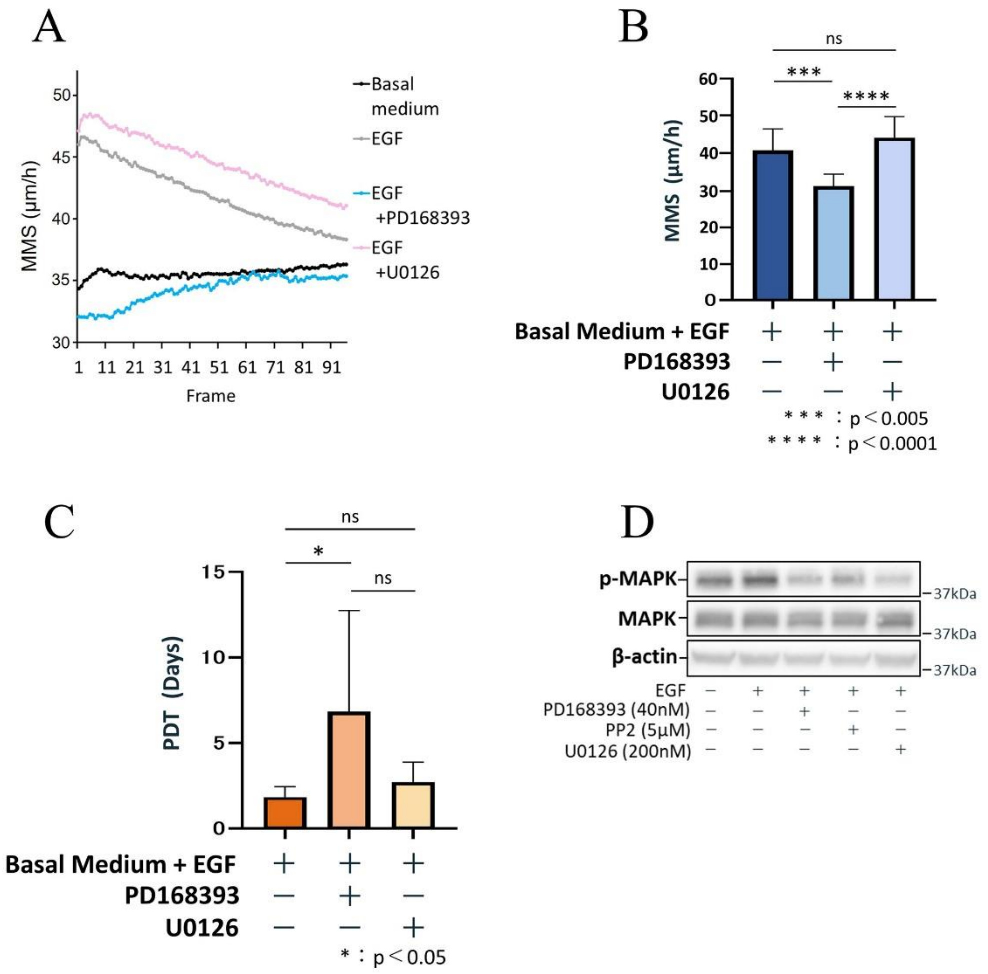

Supplementary Figure 2: U0126, a specific MEK1/2 inhibitor, affects oral keratinocyte cell motility and proliferative capacity.

(A) Changes in the MMS (mean motion speed, an index of cell motility: N = 10) over a period of 24 h consisting of 96 frames. Cells were cultured in a basal medium without EGF, a basal medium containing 1 ng/mL EGF, and a basal medium containing 1 ng/mL EGF and either 40 nM PD168393 or 200 nM U0126.

(B) The mean values of MMS for all 96 frames are shown to compare the motility of cells cultured in a basal medium containing 1 ng/mL EGF, and a basal medium containing 1 ng/mL EGF and either 40 nM PD168393 or 200 nM U0126. Data are shown as the mean ± SD. Significant differences among the groups were determined by one-way analysis of variance with Tukey's post hoc tests. ***$p < 0.005$, and ****$p < 0.0001$.

(C) Mean values of PDT (population doubling time, an index of proliferative capacity: N = 10) are shown to compare the PDT of cells cultured in a basal medium containing 1 ng/mL EGF, and a basal medium containing 1 ng/mL EGF and either 40 nM PD168393 or 200 nM U0126. Data are shown as the mean ± SD. Significant differences among the groups were determined by one-way analysis of variance with Tukey's post hoc tests. *$p < 0.05$.

(D) Representative immunoblot images for proteins involved in MAPK signaling are shown. Cells were cultured in a basal medium containing 1 ng/mL EGF, and a basal medium containing 1 ng/mL EGF and either 40 nM PD168393, 5 μM PP2 or 200 nM U0126.
