# Supplementary material for: The EGF/EGFR axis and its downstream signaling pathways regulate the motility and proliferation of cultured oral keratinocytes
Source: FEBS Open Bio. 2023 Jun 4;13(8):1469–84. doi: 10.1002/2211-5463.13653 (PMC10392064; doi:10.1002/2211-5463.13653)
Supplement: Supplementary file 3 — Fig. S3. PF‐562271, a specific FAK inhibitor, affects oral keratinocyte cell motility and proliferative capacity. (A) Changes in the MMS (mean motion speed: an index of cell motility: N = 10) over a period of 24 h consisting of 96 frames. Cells were cultured in a basal medium without EGF, a basal medium containing 1 ng·mL−1 EGF, and a basal medium containing 1 ng·mL−1 of EGF and either 40 nm PD168393 or 3 μm PF‐562271. (B) The mean values of MMS for all 96 frames are shown to compare the motility of cells cultured in a basal medium with 1 ng·mL−1 of EGF and a basal medium containing 1 ng·mL−1 of EGF and either 40 nm PD168393 or 3 μm PF‐562271. Data are shown as the mean ± SD. Significant differences among the groups were determined by one‐way ANOVA with Tukey's post hoc tests. *P < 0.05. (C) Mean values of PDT (population doubling time: an index of proliferative capacity: N = 10) are shown to compare the PDT of cells cultured in a basal medium with 1 ng·mL−1 EGF and a basal medium containing 1 ng·mL−1 of EGF and either 40 nm PD168393 or 3 μm F‐562271. Data are shown as the mean ± SD. Significant differences among the groups were determined by one‐way ANOVA with Tukey's post hoc tests. *P < 0.05. (D) Representative immunoblot images for proteins involved in FAK signaling are shown. Cells were cultured in a basal medium containing 1 ng·mL−1 of EGF and a basal medium containing 1 ng·mL−1 of EGF and either 40 nm PD168393, 2 μm LY294002, 5 μm PP2, or 3 μm PF‐562271. [file FEB4-13-1469-s009.pdf]

Supplementary Figure 3

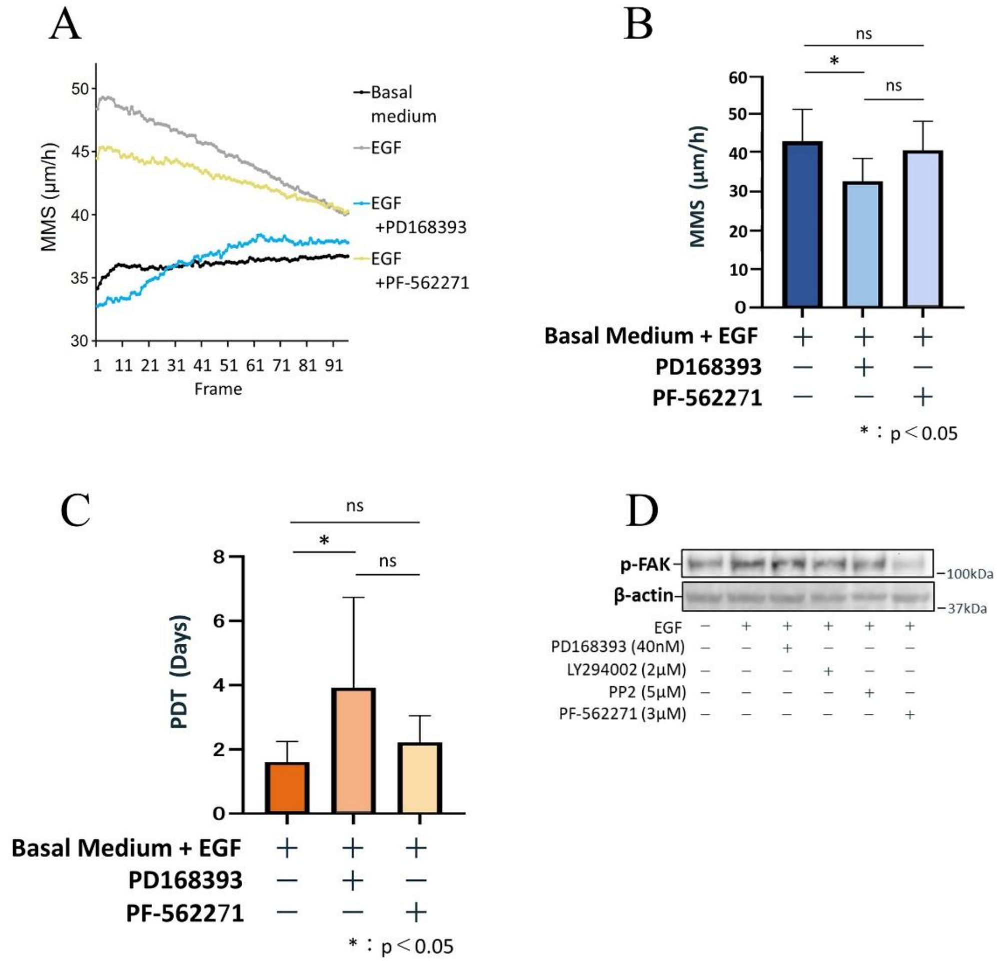

Supplementary Figure 3: PF-562271, a specific FAK inhibitor, affects oral keratinocyte cell motility and proliferative capacity.

(A) Changes in the MMS (mean motion speed: an index of cell motility: N = 10) over a period of 24 h consisting of 96 frames. Cells were cultured in a basal medium without EGF, a basal medium containing 1 ng/mL EGF, and a basal medium containing 1 ng/mL of EGF and either 40 nM PD168393 or 3 μM PF-562271.

(B) The mean values of MMS for all 96 frames are shown to compare the motility of cells cultured in a basal medium with 1 ng/mL of EGF, and a basal medium containing 1 ng/mL of EGF and either 40 nM PD168393 or 3 μM PF-562271. Data are shown as the mean ± SD. Significant differences among the groups were determined by one-way analysis of variance with Tukey's post hoc tests. $*p < 0.05$.

(C) Mean values of PDT (population doubling time: an index of proliferative capacity: N = 10) are shown to compare the PDT of cells cultured in a basal medium with 1 ng/mL EGF, and a basal medium containing 1 ng/mL of EGF and either 40 nM PD168393 or 3 μM F-562271. Data are shown as the mean ± SD. Significant differences among the groups were determined by one-way analysis of variance with Tukey's post hoc tests. $*p < 0.05$.

(D) Representative immunoblot images for proteins involved in FAK signaling are shown. Cells were cultured in a basal medium containig 1 ng/mL of EGF, and a basal medium containing 1 ng/mL of EGF and either 40 nM PD168393, 2 μM LY294002, 5 μM PP2 or 3 μM PF-562271.
